# Enantioselective Separation of 4,8-DHT and Phytotoxicity of the Enantiomers on Various Plant Species

**DOI:** 10.3390/molecules21040528

**Published:** 2016-04-22

**Authors:** Li Yang, Xiao-Yan Ma, Xiao Ruan, De-An Jiang, Cun-De Pan, Qiang Wang

**Affiliations:** 1 Ningbo Institute of Technology, Zhejiang University, Ningbo 315100, China; yangli0817@yeah.net (L.Y.); 15676230509@yeah.net (X.-Y.M.); ruanxiao@nit.net.cn (X.R.); 2 College of Life Sciences, Zhejiang University, Hangzhou 310058, China; dajiang@zju.edu.cn; 3 College of Forestry and Horticulture, Xinjiang Agricultural University, Urumqi 830052, China; pancunde@163.com

**Keywords:** 4,8-dihydroxy-1-tetralone (4,8-DHT), chiral-selective separation, enantiomers, phytotoxicity

## Abstract

As a candidate for bioherbicide, 4,8-dihydroxy-1-tetralone (4,8-DHT) was isolated from *Caryospora callicarpa* epicarp and its two enantiomers, *S*-(+)-isosclerone and *R*-(−)-regiolone, were separated by chiral high-performance liquid chromatography (HPLC) on a Chiralcel OD column with chiral stationary phase (CSP)-coated cellulose-tris(3,5-dimethylphenylcarbamate). Then, the phytotoxicity of 4,8-DHT and its enantiomers toward the seeds germination and seedling growth of the five tested plant species, including lettuce (*Latuca sativa*), radish (*Raphanus sativus*), cucumber (*Cucumis sativus*), onion (*Allium cepa*), and wheat (*Triticum aestivum*), were investigated and the results indicated a hormesis at low concentration of 4,8-DHT and its enantiomers, but a retardant effect at high concentration. Between the two enantiomers of 4,8-DHT, the *S-*(+)-isosclerone was more toxic to seeds germination and seedling growth of the five tested plant species than the *R*-(−)-regiolone, and also the phytotoxicity of *S*-(+)-isosclerone varied with different plants. For example, *S*-(+)-isosclerone was the most active to seedling growth of lettuce, indicating that *S*-(+)-isosclerone had specific effects on different organisms. Thus, all of the chirality and concentration of 4,8-DHT, as well as the affected plant species, need to be taken into consideration in the development and utilization of 4,8-DHT.

## 1. Introduction

Chirality of a pair of molecules with a non-superposable mirror image is almost caused by the presence of an asymmetric carbon atom. Chiral molecules are widely used as the mainstay of pesticides. As many as 25% of all pesticide active ingredients are chiral, existing as two mirror images called enantiomers [1]. For economic reasons, chiral pesticides are primarily used as a mixture of enantiomers or racemates [2]. *In vivo*, although the enantiomers of chiral pesticides have identical physical and chemical properties, they usually display different physiochemical and biochemical properties in metabolism, excretion, side effects, and toxicity, even acting as an antagonist [3,4,5]. One enantiomer of a chiral pesticide may have the desired effects on a target species, whereas the other enantiomer may not [2]. In recent years, the enantioselective behavior of chiral pesticides has received more attention at the forefront of chemistry and toxicology research [6,7,8]. It was reported that the enantiomers of many chiral pesticides showed different activity. For example, the toxicity to *Daphnia* revealed that the (−)-enantiomer of leptophos showed a lower toxicity than its (+)-form and racemic form [9]; (+)-fenamiphos proved be about 20 times more toxic to *Daphnia* than (−)-fenamiphos [10]; the *R*-(+)-enantiomer of the herbicide diclofop-methyl showed significantly higher herbicidal activity than the *S*-(−)-enantiomer [11]; and the (2*S*,3*S*)-(−)-enantiomer of paclobutrazol was more active than the (2*R*,3*R*)-(+)-enantiomer toward wheat seedling shoot or root growth inhibition [8]. Moreover, the enantioselectivity occurred in aquatic toxicity for chiral compound benalaxyl and *S*-(+)-benalaxyl was less toxic than *R*-(+)-benalaxyl and its racemate to freshwater microalgae *Scenedesmus obliquus* [12], and the significant enantioselective toxicity of imazapyr in *Arabidopsis thaliana* for greater toxicity with (+)-imazapyr than (+)-imazapyr and (−)-imazapyr, suggesting that (+)-imazapyr had a more herbicidal effect [13]. Therefore, research on stereoselective behaviors of such compounds is necessary to promote the use of pure active enantiomers in order to reduce waste and undesirable effects.

In recent years, signficant progress has been made for the separation of chiral pesticides. Among the many types of chiral separation methods, high-performance liquid chromatography (HPLC) with a chiral stationary phase column (CSP column) and a specific mobile phase has been used most widely for racemic resolution. HPLC is preferable to separate enantiomers because of its robust enantioselectivity and applicability on both the analytical and the preparative scale [14]. Many of the commercial pesticides used as racemates in agricultural practice can be separated by HPLC [9,15,16,17].

4,8-DHT, as a natural product, was first isolated from a *Scytalidium* species [18]. Subsequently, the dihydroxy naphthalenone has been found in both enantiomeric forms (named as regiolone and isosclerone) in a number of fungi and plants [19,20,21]. Biological properties of 4,8-dihydroxy-1-tetralone have been largely studied, including cytotoxic activities against human cancer cells [20,22,23]. It was found that 4,8-DHT isolated and purified from *Caryospora callicarpa* epicarp could significantly inhibit the germination of lettuce and cucumber and decrease the radicle length, plumule length, and fresh weight of lettuce and onion seedlings [21]. Based on our previous research, 4,8-DHT, as an allelochemical, has the potential to become a natural herbicide [21,24]. In view of its molecular structure, 4,8-DHT has an asymmetric carbon atom, typically consisting of two enantiomers. However, very little information is available on the phytotoxicity of 4,8-DHT to plants and other non-target organisms. Therefore, this study aimed to understand the toxicity of 4,8-DHT and its enantiomers to the plants at the morphological level. We first developed a separation method by using HPLC equipped with a chiral column to prepare two enantiomers of 4,8-DHT, and then comprehensively assessed the phytotoxicity of the 4,8-DHT racemate and its enantiomers on five plant species, including lettuce (*Latuca sativa*), radish (*Raphanus sativus*), cucumber (*Cucumis sativus*), onion (*Allium cepa*), and wheat (*Triticum aestivum*).

## 2. Results and Discussion

### 2.1. Separation of 4,8-DHT Enantiomers

Enantiomeric separation of 4,8-DHT by HPLC on the Chiralcel OD column with CSP-coated cellulose-tris(3,5-dimethylphenylcarbamate) could be easily performed. Among phenylcarbamate derivatives, 3,5-dimethylphenylcarbamate affords the most useful CSP, which can separate a wide range of racemates [25], and in view of the structure of 4,8-DHT containing a C=O group, phenyl ring, and hydroxyl directly linking to the chiral center, the 3,5-dimethylphenylcarbamate was selected as the CSP.

**Figure 1:**
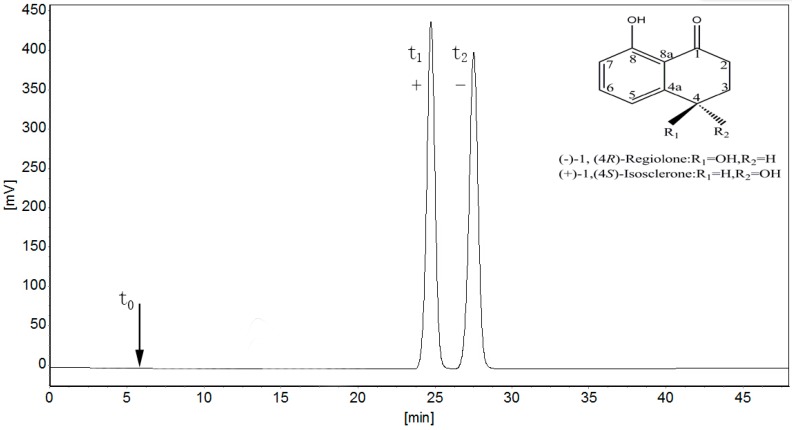
The elution order of 4,8-DHT enantiomers. *Conditions*: mobile phase, *n*-hexane/iso-propanol/AA (95:5:0.1, *v*/*v*/%); flow rate, 0.8 mL·min^−1^; column temperature, 20 °C.

Alcohol in the mobile phase competes for chiral bonding sites with chiral solutes and can alter the steric environment of the chiral cavities on the CSP by binding to achiral sites at or near the chiral cavity [26]. There have been many studies to change the polarity of the mobile phase with various alcohols, such as *n*-butanol, *iso*-butanol, *n*-propanol, *iso*-propanol, and ethanol [27,28,29]. Considering 4,8-DHT is a weak acidic material [30], a certain amount of acetic acid (AA) is added into mobile phase, which can reduce the tailed of chromatographic peaks and increase the symmetry of peaks. Thus, in this study, the optimized chromatographic condition was determined to be *n*-hexane/*iso*-propanol (95:5, *v*/*v*) as the mobile phase containing 0.1% AA with a flow rate of 0.8 mL·min^−1^ at 20 °C and UV detection at 254 nm. The capacity factor of regiolone and isosclerone (*k*_1_ and *k*_2_), the separation factor (α), and the resolution (*Rs*) were calculated as follow: *k*_1_ = 4.09, *k*_2_ = 4.77, α = 1.16, *Rs* = 1.70. The elution order of 4,8-DHT enantiomers was distinguished at 254 nm of optical rotation detection. The first eluted enantiomer was *S*-(+)-isosclerone at 24.4 min and the second was *R*-(−)-regiolone at 27.7 min (Figure 1). Additionally, a 20.8 mg 4,8-DHT sample was separated through preparative HPLC, and the elution of enantiomers were collected. The contents of *S*-(+)-isosclerone and *R*-(−)-regiolone were 10.2 mg (yield 49.0%) and 10.0 mg (48.2%).

### 2.2. Effects of Rac-4,8-DHT, S-(+)-Isosclerone, and R-(−)-Regiolone on Germination of the Tested Plants

Crop seeds are commonly selected for use in phytotoxic bioassays because they satisfy a number of selection criteria: readily available, affordable, repeatable and reliable; and they germinate quickly, completely, and uniformly [18]. In this study, we selected five crops as test species. The enantioselective toxicity of 4,8-DHT on plants can be estimated by several indices. Germination vigor and germination rate are two important indices to determine the seeds’ germination [21,31]. In this study, rac-4,8-DHT, *S*-(+)-isosclerone, and *R*-(−)-regiolone on germination of the tested plants were measured in terms of these parameters (Table 1). Compared with the control, germination of the five tested plants was significantly affected after they were exposed to different concentrations of rac-4,8-DHT, *S*-(+)-isosclerone, and *R*-(−)-regiolone (Table 1). For lettuce, there was a pronounced hormesis to the germination vigor when exposed to the racemate.

*S*-(+)-isosclerone and *R*-(−)-regiolone at the 2 mM level, whereas the germination vigor was lower than the control at high concentration of 4,8-DHT and its enantiomers, and the retardant effect of *S*-(+)-isosclerone on the germination vigor was higher than that of the racemate or *R*-(−)-regiolone. Regarding to the germination rate of lettuce exposed to 4,8-DHT and its enantiomers, a similar pattern was displayed. For cucumber, a pronounced hormesis on the germination vigor appeared after treatment with 4,8-DHT and its enantiomers at the concentration of 1–3 mM except for 1 mM *R*-(−)-regiolone, and the germination vigor was at 2 and 3 mM levels of 4,8-DHT and its enantiomers, except for 2 mM *R*-(−)-regiolone. After treatment with the highest concentration of 4,8-DHT and its enantiomers (4 mM), the germination rate was inhibited, compared with the control, and *S*-(+)-isosclerone caused the most inhibition, whereas only *S*-(+)-isosclerone had an inhibitory effect on germination vigor. After exposure to 4,8-DHT and its enantiomers, the 1–3 mM concentrations had no effect on the germination rate of radish, except for 3 mM *S*-(+)-isosclerone. In addition, the highest concentration (4 mM) had a retardant effect, and the most retardant effect was observed in *S*-(+)-isosclerone. The hormesis on the germination vigor of radish induced by *S*-(+)-isosclerone at 1–3 mM was higher than that by the racemate and *R*-(−)-regiolone. The germination rate of radish exposed to the highest concentration of 4,8-DHT and its enantiomers was similar to the germination vigor of radish. For the germination of onion and wheat, the hormesis was only observed at the lowest concentration of 4,8-DHT and its enantiomers. The hormesis on onion was significantly different among 4,8-DHT and its enantiomers, in the order of *S*-(+)-isosclerone > racemate > *R*-(−)-regiolone for germination vigor, and the germination rate exposed to *S*-(+)-isosclerone and racemate was higher than *R*-(−)-regiolone. For wheat, only *R*-(−)-regiolone had a significant hormesis on the germination vigor and germination rate.

The germination vigor and rate of onion and wheat incubated with 2–4 mM of 4,8-DHT and its enantiomers were significantly decreased, especially at 4 mM, the inhibition rate of germination vigor and rate was the highest. At the concentration of 2 mM, *S*-(+)-isosclerone inhibited germination vigor and germination rate in wheat more than the racemate and *R*-(−)-regiolone. In addition, the germination rate in onion treated by 3 mM and 4 mM of *S*-(+)-isosclerone were significantly more than that of the racemate and *R*-(−)-regiolone. The results of the seeds germination indicated that there was a significant toxicity of rac-4,8-DHT, *S*-(+)-isosclerone, and *R*-(−)-regiolone to the five plants. Furthermore, the phytotoxicity of rac-4,8-DHT, *S*-(+)-isosclerone, and *R*-(−)-regiolone was found to be dependent on their concentration and chirality.

### 2.3. Effects of Rac-4,8-DHT, S-(+)-Isosclerone, and R-(−)-Regiolone on Seedlings Growth of the Tested Plants

**Figure 2:**
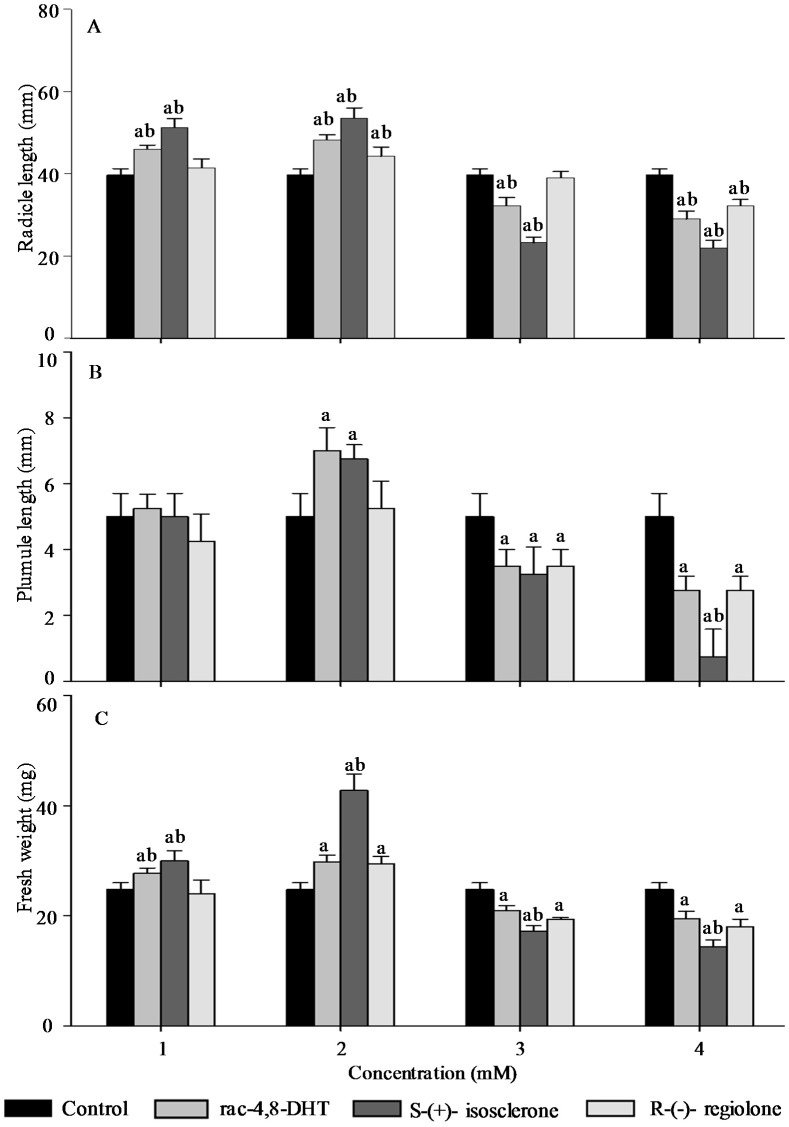
Effect of rac-4,8-DHT, *S*-(+)-isosclerone, and *R*-(−)-regiolone at different concentrations on radicle length (**A**); plumule length (**B**); and fresh weight (**C**) in lettuce. a denotes a significant difference between control and treatments (*p* < 0.05); b denotes significant difference among rac-4,8-DHT, *S*-(+)-isosclerone, and *R*-(−)-regiolone (*p* < 0.05).

**Figure 3:**
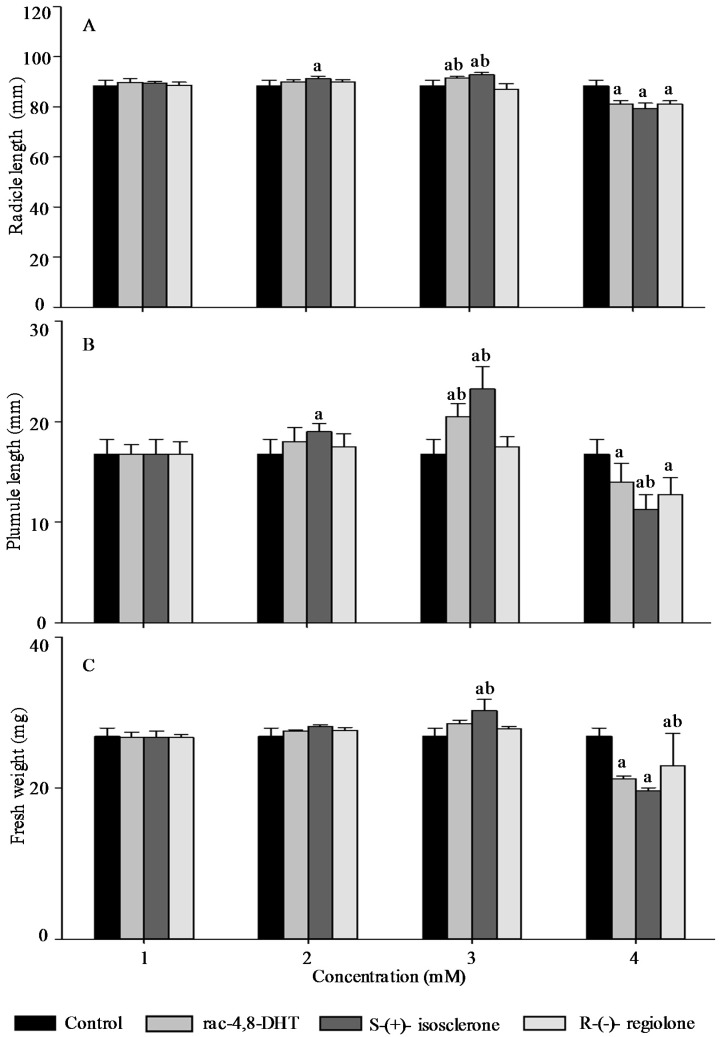
Effect of rac-4,8-DHT, *S*-(+)-isosclerone, and *R*-(−)-regiolone at different concentrations on radicle length (**A**); plumule length (**B**); and fresh weight (**C**) in cucumber. a denotes a significant difference between control and treatments (*p* < 0.05); b denotes a significant difference among rac-4,8-DHT, *S*-(+)-isosclerone, and *R*-(−)-regiolone (*p* < 0.05).

**Figure 4:**
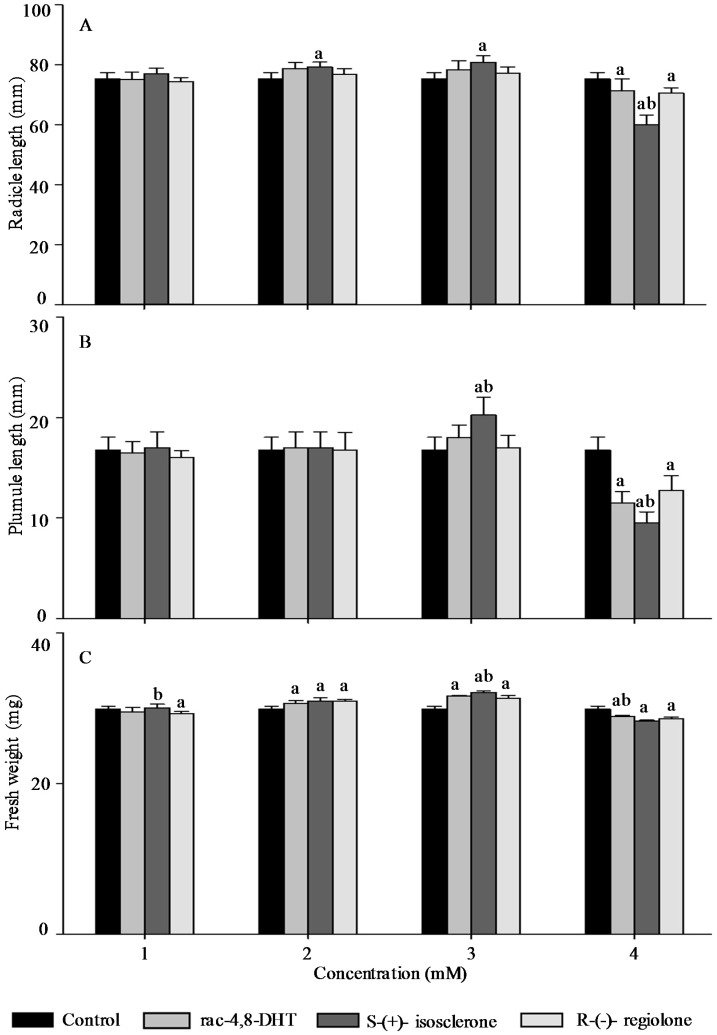
Effect of rac-4,8-DHT, *S*-(+)-isosclerone, and *R*-(−)-regiolone at different concentrations on radicle length (**A**); plumule length (**B**); and fresh weight (**C**) in radish. a denotes a significant difference between control and treatments (*p* < 0.05); b denotes a significant difference among rac-4,8-DHT, *S*-(+)-isosclerone and *R*-(−)-regiolone (*p* < 0.05).

**Figure 5:**
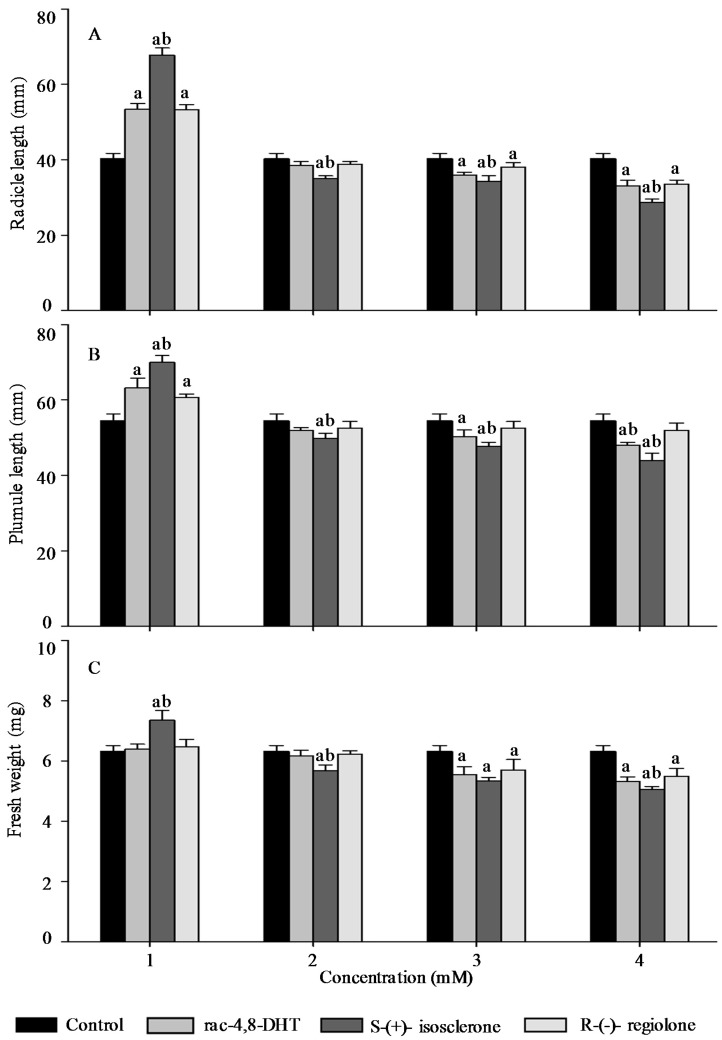
Effect of rac-4,8-DHT, *S*-(+)-isosclerone, and *R*-(−)-regiolone at different concentrations on radicle length (**A**); plumule length (**B**); and fresh weight (**C**) in onion. a denotes significant difference between control and treatments (*p* < 0.05); b denotes significant difference among rac-4,8-DHT, *S*-(+)-isosclerone and *R*-(−)-regiolone (*p* < 0.05).

**Figure 6:**
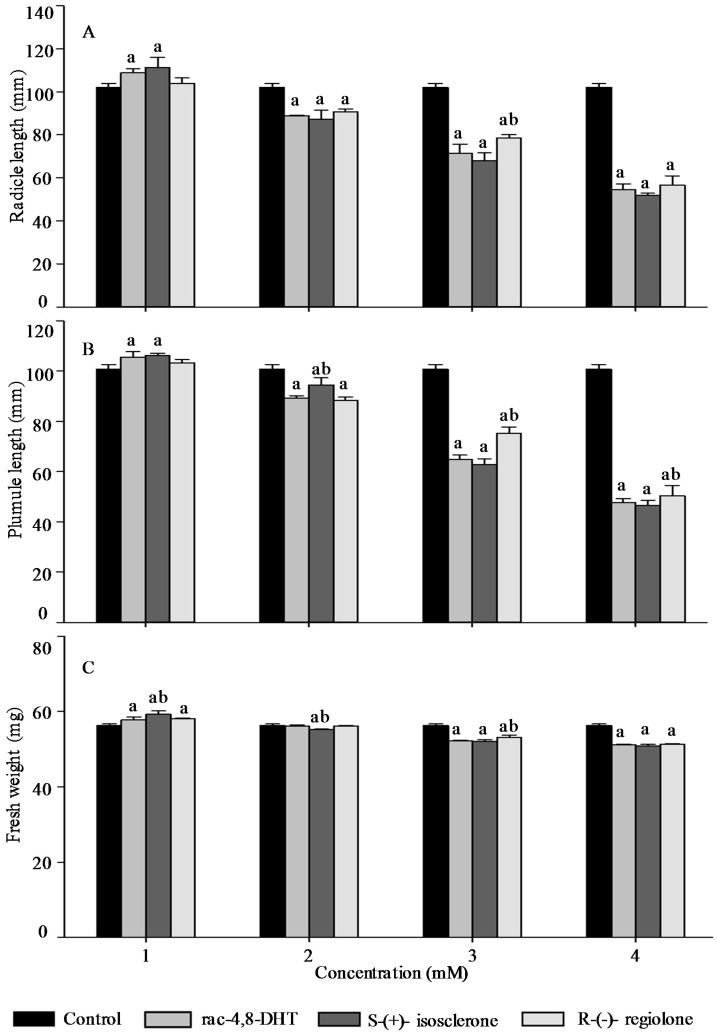
Effect of rac-4,8-DHT, *S*-(+)-isosclerone, and *R*-(−)-regiolone at different concentrations on radicle length (**A**); plumule length (**B**); and fresh weight (**C**) in wheat. a denotes a significant difference between control and treatments (*p* < 0.05); b denotes a significant difference among rac-4,8-DHT, *S*-(+)-isosclerone and *R*-(−)-regiolone (*p* < 0.05).

Radicle length, pumule length, and fresh weight are important traits for measuring plant growth [18,26] and, thus, these parameters were used to determine enantioselective activities of 4,8-DHT and its enantiomers in the five tested plants’ seedlings. As shown in Figure 2, Figure 3, Figure 4, Figure 5 and Figure 6, the growth trends of the plants seedlings exposed to different concentration of rac-4,8-DHT, *S*-(+)-isosclerone, and *R*-(−)-regiolone were similar to those of the germination in the tested plants. There was a hormesis on radicle length, pumule length, and fresh weight when exposed to 4,8-DHT and its enantiomers at concentrations of 1–2 mM for lettuce, at 1–3 mM for cucumber and radish, and at the lowest concentration of 1 mM for onion and wheat, while the retardant effect was observed at concentrations of 3–4 mM for lettuce, at 2–4 mM for onion and wheat, and at the highest concentration of 4 mM for cucumber and radish, respectively. These results were consistent with the previous observations [8,13], in where paclobutrazol and its enantiomers at low concentrations of 0.125–0.5 mg·L^−1^ induced hormesis on root growth of rice seedlings, whereas high concentration had retardant effects [8]. The enantioselective toxicity of imazapyr on *Arabidopsis thaliana* showed that the lowest concentration of herbicides had no effect on plant shoots, but the shoots exposed to a high concentration of (+)-imazapyr were smaller than those exposed to (−)-imazapyr and (±)-imazapyr [13]. Thus, the toxic effect of 4,8-DHT and its enantiomers on the growth of the five tested plants seedlings was related to their concentration.

It has been reported that enantioselective effect of paclobutrazol on *Microcystis aeruginosa* and *Anabaena* sp. was different. The (2*S*,3*S*)-(−)-enantiomer showed stronger stimulatory activity on *M. aeruginosa* cyanobacteria than the (2*R*,3*R*)-(+)-enantiomer, whereas the latter was a more potent stimulator of *Anabaena* sp. growth, indicating that enantioselectivity has specific effects on different organisms [8]. However, in this study, compared with the parameters of seedlings growth between 4,8-DHT and its enantiomers, we found that 4,8-DHT and its enantiomers could improve the quality of the five tested plants seedling at low concentration, while pure *S*-(+)-isosclerone was more effective than the others. Therefore, pure *S*-(+)-isosclerone can be used as a regulator of plant growth to reduce the environmental load. Meanwhile, *S*-(+)-isosclerone also inhibited seedlings growth of the five tested plants more than *R*-(−)-regiolone at the higher concentration. This result was paralleled to toxicity of the chiral herbicide diclofop-methyl and its enantiomers, in where *S*-(−)-enantiomer of diclofop-methyl had a stronger toxicity to both *Chlorella ulgaris* and *Scenedesmus obliquus* than the *R*-(+)-enantiomer [32]. It is clear that the toxicity of 4,8-DHT and its enantiomers has specific effects on different organisms, but the mechanism for differential enantiomers specific responses in these species is uncertain and deserves further research. Among the five tested plants, seedling growth of lettuce was the most sensitive to *S*-(+)-isosclerone treatments. At the treatment concentration of 1 mM the radicle length and fresh weight of lettuce treated with *S*-(+)-isosclerone were 26.8% and 25.0% higher than those of lettuce treated with *R*-(−)-regiolone, respectively. At the treatment concentration of 2 mM *S*-(+)-isosclerone enhanced the fresh weight of lettuce by 44.8% higher than the racemate and *R*-(−)-regiolone. In addition, the radicle length and fresh weight of lettuce treated by 4 mM *S*-(+)-isosclerone were 21.8% and 26.3% lower than those of lettuce treated by the 4 mM racemate and *R*-(−)-regiolone, respectively. However, the plumule length of lettuce cultivated with all different groups showed no significant differences between the *S*-(+)-isosclerone and *R*-(−)-regiolone treatment, except for *S*-(+)-isosclerone at the higher concentration level of 4 mM. Different enantiomer-specific responses in radicle and plumule length have also been studied for other chiral herbicides [33,34]. For instance, (2*S*,3*S*)-paclobutrazol reduced the shoot growth more effectively than the root growth, while (2*R*,3*R*)-paclobutrazol reduced the root growth more effectively than the shoot growth [33]. The R-enantiomer of diclofop acid was more active toward root growth while the *S*-enantiomer was more active toward leaf growth [34]. However, the mechanism is still uncertain and requires further research in the future. As indicated by the above results for the five tested plants, all of the factors including concentration and configuration forms of 4,8-DHT, as well as the plant species, must be considered in order to estimate the toxic behaviors of 4,8-DHT.

## 3. Materials and Methods

### 3.1. Chemicals, Plant Materials and Reagents

4,8-DHT was isolated and purified from *C. callicarpa* epicarp in our laboratory (purity ≥ 98%). 1,3,5-tri-*tert*-butylbenzene was purchased from Sigma Aldrich (St. Louis, MO, USA). Seeds of lettuce (*Latuca sativa*), radish (*Raphanus sativus*), cucumber (*Cucumis sativus*), onion (*Allium cepa*), and wheat (*Triticum aestivum*) were purchased from the market in Ningbo, China, and used for bioassay. HPLC grade of *iso*-butanol, *n*-butanol, *iso*-propanol, *n*-propanol, and ethanol were purchased from Tedia Company Inc. (Fairfield, IA, USA). All other chemicals and solvents in analytical grade were purchased from commercial sources.

### 3.2. Chromatographic Separation and Analysis

Analytical HPLC: Enantioseparation of 4,8-DHT was conducted on the HPLC system consisting of two LC-10AT HPLC pumps, an SPD-10A UV detector monitored at 254 nm, and an FCV-12AH 6-PortValve, controlled by a CBM-10A module (all items obtained from Shimadzu, Duisburg, Germany). Column temperature was controlled by an AT-930 heater column attemperator (Tianjin Automatic Science Instrument, Tianjin, China). The column was a Chiralcel OD column (250 mm × 4.6 mm, 5 μm) (Guangzhou Research and Creativity Biotechnology Co. Ltd., Guangzhou, China) with chiral stationary phase (CSP)-coated cellulose-tris(3,5-dimethylphenylcarbamate). The mobile phase consisted of a mixture of *n*-hexane and *iso*-propanol (95:5, *v/v*) containing 0.1% acetic acid (AA) under a flow rate of 0.8 mL·min^−1^ at 20 °C. The injection of samples was performed using a 7725i manual injector and the sample injection volume was 20 μL.

Preparative HPLC: The column was Chiralcel OD column (250 mm × 20 mm, 5 μm) (Guangzhou Research and Creativity Biotechnology Co. Ltd., Guangzhou, China). The mobile phase consisted of a mixture of *n*-hexane and *iso*-propanol (90:10, *v/v*) monitored at 254 nm under a flow rate of 12 mL·min^−1^ at 20 °C. The sample injection volume was 5 mL.

### 3.3. Effects of 4,8-DHT on Seed Germination

One hundred grains of surface-sterilized lettuce seeds were placed in each sterile Petri dish (15 cm diameter) lined with two layers of filter paper. Then, 10 mL of various concentrations of racemate, *S*-(+)-isosclerone, or *R*-(−)-regiolone (1, 2, 3, and 4 mM), and distilled water as a control, were added into each Petri dish, respectively. Seeds were incubated in an artificial intelligence simulation incubator under an 8/16 h (day/night) photo period with a photon flux density of 40 µmol·m^−2^·s^−1^ at a day/night temperature of 25/15 °C. Experiments of seed germination were conducted according to ISTA [35]. Germination (radicle emergence) was measured at four and seven days after treatment for lettuce, four and 10 days for radish, four and eight days for cucumber and wheat, and six and 12 days for onion.

### 3.4. Effects of 4,8-DHT on Seedling Growth

Pre-germination of the tested seeds were preceded in plastic boxes (20 × 15 × 10 cm) lined with two layers of filter paper for 3–4 days until radicle emergence. One hundred successfully-germinated seeds were placed in Petri dishes and then 10 mL of various concentrations of racemate, *S*-(+)-isosclerone, or *R*-(−)-regiolone (1, 2, 3, and 4 mM), and distilled water as a control, were added into the dishes. Seedlings were incubated in an artificial intelligence simulation incubator (incubation conditions were the same as seed germination). Five seeds were randomly removed, and the length of the plumule and radicle were measured with a vernier caliper (GB/T 1214.2-1996, Measuring Instrument Ltd., Shanghai, China). The fresh weight of seedlings was also recorded (Mettler Toledo Instrument Ltd., Boston, MA, USA). The plumule length, radicle length, and fresh weight of each tested seedlings were measured at 10 days after incubation.

### 3.5. Statistic Analysis

All the above tests and measurements were performed in four replicates. All data were statistically analyzed using Sigmaplot software. Values were considered significant when the results of one-way ANOVA using least significant difference showed *p* < 0.05 using SPSS software. All other values in the text are presented as the mean ± SD.

## 4. Conclusions

In this study, enantiomers of 4,8-DHT were effectively separated by HPLC on the Chiralcel OD column with CSP-coated cellulose-tris(3,5-dimethylphenylcarbamate) and showed different toxicity towards seed germination and seedling growth of the five tested plant species. Low concentrations of racemate, *S*-(+)-isosclerone, and *R*-(−)-regiolone induced hormesis on seed germination and seedling growth of the tested plant species, whereas high concentrations had a retardant effect. By comparison, pure *S*-(+)-isosclerone was more toxic to seed germination and seedling growth of the five plants and, thus, could be potentially applied as a herbicide to reduce the environmental load. Among the five tested plants, seedling growth of lettuce was most sensitive to *S*-(+)-isosclerone treatments. Additionally, it is clear that *S*-(+)-isosclerone has specific effects on different organisms. These results indicated that the toxic effects of 4,8-DHT in the five tested plants were different, and all of the concentrations and configuration forms of 4,8-DHT, as well as plants species, must be taken into consideration in order to develop and utilize 4,8-DHT.

## Figures and Tables

**Table 1:** Effect of rac-4,8-DHT, *S*-(+)-isosclerone, and *R*-(−)-regiolone at different concentrations on seed germination of five plant species.

Plant Species	Forms	Germination Vigor					Germination Rate				
		Control	1 mM	2 mM	3 mM	4 mM	Control	1 mM	2 mM	3 mM	4 mM
Lettuce	rac	75 ± 2	76 ± 1	83 ± 1 ^a^	69 ± 2 ^a^	60 ± 1 ^a^	85 ± 1	86 ± 1	94 ± 1 ^a^	83 ± 2 ^a^	79 ± 1 ^a^
	*S*		76 ± 2	85 ± 2 ^a^	65 ± 2 ^a,b^	57 ± 2 ^a,b^		87 ± 1	96 ± 1 ^a^	82 ± 2 ^a^	62 ± 1 ^a,b^
	*R*		76 ± 2	82 ± 2 ^a^	69 ± 2 ^a^	63 ± 2 ^a^		86 ± 2	92 ± 1 ^a^	83 ± 2 ^a^	80 ± 1 ^a^
Cucumber	rac	83 ± 1	85 ± 1 ^a^	86 ± 2 ^a^	90 ± 2 ^a^	81 ± 2	87 ± 2	88 ± 2	90 ± 1 ^a^	93 ± 2 ^a^	82 ± 2 ^a^
	*S*		86 ± 2 ^a^	87 ± 2 ^a^	90 ± 2 ^a^	79 ± 2 ^a^		89 ± 1	90 ± 1 ^a^	93 ± 2 ^a^	80 ± 2 ^a,b^
	*R*		84 ± 2	86 ± 1 ^a^	89 ± 1 ^a^	81 ± 1		88 ± 2	88 ± 1	91 ± 1 ^a,b^	83 ± 1 ^a^
Radish	rac	82 ± 1	83 ± 1	83 ± 1	86 ± 2 ^a^	79 ± 1 ^a^	88 ± 2	87 ± 2	89 ± 1	91 ± 1	83 ± 2 ^a^
	*S*		84 ± 1 ^a^	84 ± 2 ^a^	89 ± 1 ^a,b^	74 ± 1 ^a,b^		88 ± 2	90 ± 1	93 ± 1 ^a,b^	77 ± 2 ^a,b^
	*R*		82 ± 1	83 ± 1	85 ± 1 ^a^	80 ± 2 ^a^		88 ± 1	90 ± 1	90 ± 2	82 ± 2 ^a^
Onion	rac	40 ± 4	63 ± 3 ^a,b^	36 ± 3	34 ± 2 ^a^	32 ± 1 ^a^	45 ± 4	68 ± 1 ^a,b^	43 ± 1	42 ± 1 ^a^	40 ± 1 ^a^
	*S*		67 ± 4 ^a,b^	33 ± 2 ^a^	32 ± 2 ^a^	30 ± 1 ^a^		69 ± 3 ^a,b^	40 ± 1 ^a,b^	39 ± 1 ^a^	33 ± 1 ^a,b^
	*R*		59 ± 2 ^ab^	36 ± 2 ^a^	34 ± 2 ^a^	33 ± 2 ^a^		63 ± 1 ^a^	43 ± 1	42 ± 1 ^a^	40 ± 1 ^a^
Wheat	rac	68 ± 2	71 ± 2	64 ± 2	38 ± 5 ^a^	27 ± 2 ^a^	80 ± 2	83 ± 2	75 ± 4 ^a^	43 ± 5 ^a^	27 ± 2 ^a^
	*S*		74 ± 3^a^	58 ± 3 ^a,b^	35 ± 4 ^a^	24 ± 3 ^a^		85 ± 1 ^a^	65 ± 1 ^a,b^	40 ± 4 ^a^	25 ± 1 ^a^
	*R*		70 ± 1	65 ± 1	39 ± 3 ^a^	28 ± 3 ^a^		83 ± 1	79 ± 3	50 ± 4 ^a,b^	29 ± 1 ^a^

^a^ denotes a significant difference between control and treatments (*p* < 0.05); ^b^ denotes a significant difference among rac-4,8-DHT, *S*-(+)-isosclerone, and *R*-(−)-regiolone (*p* < 0.05).

## Abbreviations

The following abbreviations are used in this manuscript:

4,8-DHT: 4,8-dihydroxy-1-tetralone
HPLC: high-performance liquid chromatography
CSP: chiral stationary phase
AA: acetic acid
SD: standard deviation
